# Sexual dimorphism in lung transcriptomic adaptations in fetal alcohol spectrum disorders

**DOI:** 10.1186/s12931-025-03094-z

**Published:** 2025-01-08

**Authors:** Vishal D. Naik, Dylan J. Millikin, Daniel Moussa, Hong Jiang, Alexander L. Carabulea, Joseph D. Janeski, Jiahui Ding, Kang Chen, Marta Rodriguez-Garcia, Sunil Jaiman, Stephen A. Krawetz, Gil Mor, Jayanth Ramadoss

**Affiliations:** 1 Department of Obstetrics and Gynecology, C.S. Mott Center for Human Growth and Development, School of Medicine, Wayne State University, 275 E Hancock St, Rm 195, Detroit, MI 48201 USA; 2 Barbara Ann Karmanos Cancer Institute, Wayne State University, Detroit, MI USA; 3 Department of Biochemistry, Microbiology and Immunology, School of Medicine, Wayne State University, Detroit, MI USA; 4 Department of Pathology, School of Medicine, Wayne State University, Detroit, MI USA; 5 Center for Molecular Medicine and Genetics, Wayne State University, Detroit, MI USA; 6 Department of Physiology, School of Medicine, Wayne State University, Detroit, MI USA

**Keywords:** Alcohol, Pregnancy, Lung, Sexual dimorphism, Fetal

## Abstract

Current fetal alcohol spectrum disorders (FASD) studies primarily focus on alcohol’s actions on the fetal brain although respiratory infections are a leading cause of morbidity/mortality in newborns. The limited studies examining the pulmonary adaptations in FASD demonstrate decreased surfactant protein A and alveolar macrophage phagocytosis, impaired differentiation, and increased risk of Group B streptococcal pneumonia with no study examining sexual dimorphism in adaptations. We hypothesized that developmental alcohol exposure in pregnancy will lead to sexually dimorphic fetal lung morphological and immune adaptations. Pregnant rats were orogastrically treated once daily with alcohol (4.5 g/kg, gestational day [GD] 4 to 10, peak BAC, 216 mg/dl; 6.0 g/kg, GD 11 to 20, peak BAC, 289 mg/dl) or 50% maltose dextrin (isocalorically matched pair-fed controls) to control for calories derived from ethanol. Male and female fetal lung RNA from a total of 20 dams were assessed using the TapeStation (Agilent) and Qubit RNA broad-range assay. Samples with RNA Integrity Numbers (RINs) > 8 were prepared using the NEBNext Poly(A) mRNA Magnetic Isolation Module (NEB), xGen Broad-range RNA Library Prep (IDT), and xGen Normalase UDI Primer Plate 2 (IDT). Final libraries were checked for quality and quantity by Qubit hsDNA and LabChip. The samples were sequenced on the Illumina NovaSeq S4 Paired-end 150 bp. Fetal lung tissue were analyzed for histopathological assessments. Mean fetal weight, crown-rump length and placental efficiency of the alcohol-administered rats were significantly lower (*P* < 0.05) than the pair-fed control pups. Differentially expressed genes indicated a sex-linked gene regulation dichotomy with a significantly higher number of genes altered in the female fetal lungs compared to the male. Network analysis plot of downregulated genes in the females exposed to alcohol in utero showed a negative impact on T cell activation and regulation, T cell differentiation, decrease in CD8^+^ T cell number etc. The most altered genes were Cd8b, Ccl25, Cd3e, Cd27, Cd247, Cd3d, Ccr9, Cd2, Cd8a and were decreased by a log2fold change of > 2 (*P* < 0.05) in the female fetal lungs. KEGG analyses showed that male and female fetal lungs had downregulated genes associated with development and mitosis, whereas the females alone showed dysregulation of T cell genes. Comparison of gross appearance and histopathologic morphology showed that the developing lungs of both male and female fetal pups, displayed stunted differentiation, were relatively hypoplastic, and displayed a diminution of alveolar size and air spaces. Similarly, in both sexes, decreased alveolar capillarization was also evident in the alcohol-exposed fetal lungs. These data provide novel information in a growing area focused on alcohol effects on the offspring lung and its influence on appropriate fetal/neonatal immune responses and highlights the importance of examining sexual dimorphism in developmental adaptations.

## Background

Alcohol exposure during pregnancy has well documented effects on fetal development, particularly the developing brain, and the resulting spectrum of deficits is termed as fetal alcohol spectrum disorders (FASD) [1]. In the most severe form, FASD is characterized by facial dysmorphology, central nervous system deficits, and intrauterine growth restriction [2, 3]. Approximately 10% of pregnant people use alcohol across the world [4] with the highest amount estimated to be in the European region [5]. In over 40% of the countries where epidemiological studies have been conducted, over 25% drink in a binge paradigm [6], which is considered a harmful pattern of alcohol use [7]. Recent estimates in the United States place the incidence of FASD between 3.1 and 9.9% among school-aged children [8, 9]. While fetal alcohol spectrum disorder (FASD) is well known, it is important to acknowledge alcohol’s systemic effects and address gaps in knowledge regarding alcohol’s effect on fetal development to enrich our understanding of underlying pathophysiology and associated phenotypes.

There is a dearth of clinical studies examining pulmonary sequalae in neonates and children with confirmed *in utero* alcohol exposure history. Studies done in early 2000s in around 872 singleton neonates demonstrated a 2.5-fold increase in infections among infants whose mothers reported alcohol use [10]. A major effect of prenatal alcohol use on the risk of life-threatening respiratory viral infection was noted in children admitted to hospitals in Argentina [11]. A recent study done in Australian children directly correlated prenatal binge alcohol exposure and seeking medical treatment for respiratory infections [12]. In a South African cohort, infants aged 1.5–2.5 months old exhibited lower lung function following in utero alcohol use compared to unexposed infants [13]. A major gap in the respiratory studies in children is the sparce reporting of the differences between the biological sexes in infants diagnosed with FASD. Animal model-based studies are also limited in the field but they unanimously identify the developing fetal lungs as a sensitive target organ for alcohol exposure [14]; developmental alcohol exposure inhibits lung cellular growth [15], decreases lung mass and maturation [16], decreases lung VEGF expression [17], decreases surfactant production and alters surfactant content [18]. Further, FASD animal models show derangements in the innate and adaptive immune system, accompanied by decreased surfactant protein A expression [18], impaired alveolar phagocytosis [19, 20], and alveolar macrophage differentiation [21].

Both clinical and preclinical translational studies performed to date on FASD pulmonary effects form the premise for the current study. In children, the severity of respiratory infections among boys have been reported to be consistently higher than those in girls but after puberty, females are noted to exhibit more severe outcomes from respiratory infections. In fact, during pregnancy, infection with febrile human respiratory virus is directly related with more severe outcomes and hospitalization, including giving birth to low birthweight babies [22, 23]. To date, there has been no study that has investigated the effect of alcohol use in pregnancy on fetal lung development as a function of sex. We hypothesized that developmental alcohol exposure in pregnancy will lead to sexually dimorphic fetal lung morphological and immune adaptations at the level of transcriptome in a well-documented rat FASD model.

## Materials & methods

### Treatment groups and alcohol in vivo dosing paradigm

All experimental procedures were performed as per National Institutes of Health guidelines (Revised NIH Publication No. 85–23, 1996; US Department of Health, Education and Welfare, Bethesda, MD), with approval by the Animal Care and Use Committee at Wayne State University. Timed pregnant Sprague–Dawley rats purchased from Charles River (Wilmington, MA) arrived on GD 4 and were housed in a temperature-controlled room (23 °C) with a 12:12-hour light-dark cycle with ad libitum access to water. The dams were acclimatized for a day before weighing and handling. The dams were then assigned into experimental groups. Two treatment groups were utilized: (1) *a nutritional pair-fed control group (Control*,*n = 5)*, that served as a control for nutrition and for the gavage procedure. To control for the calories derived from alcohol, these pair-fed control rats were administered isocaloric maltose-dextrin (once-daily) via orogastric gavage. (2) *a binge alcohol group (Alcohol, n = 5)*, where dams were acclimatized with a once-daily gavage (orogastric) of 4.5 g/kg ethanol (22.5% weight/volume; peak blood alcohol concentration (BAC), 216 mg/dl) from gestational days (GD) 5–10, and progressed to a 6 g/kg alcohol from GD 11–19 (28.5% weight/volume; peak BAC, 289 mg/dl) [24, 25]. The exposure paradigm utilized in this study was carefully modeled after published gestational alcohol consumption patterns in humans as well as several FAS animal models [26–28]. Maternal weights were measured daily. Intake of food in the alcohol treatment group was measured every day, and an equivalent amount of food was given to the pair-fed control dams to account for additional nutritional factors. Rats were euthanized by decapitation while under isoflurane anesthesia. Immediately following euthanasia, dams were dissected, and fetuses were isolated for sex determination followed by growth measures, and tissue collection. Lungs were carefully isolated under sterile conditions and flash frozen for RNA sequencing (1 male and 1 female/dam). Lungs from a subset of the remaining pups were isolated and stored under appropriate conditions for histological analysis.

### RNA extraction and quality control

Flash frozen fetal lungs (*n* = 5 Control males, *n* = 5 Control females; *n* = 5 Alcohol males, *n* = 5 Alcohol females) were utilized for RNA isolation, quantitation and quality control assessment (University of Michigan Epigenomics Core). Frozen tissues were cryopulverized using a CP02 Cryopulverizer (Covaris). The pulverized tissue was then processed for RNA isolation using Qiagen’s DNA/RNA AllPrep kit, following the manufacturer’s instructions. RNA was eluted in 30ul of RNAse free water provided with the kit. The RNA was quantified using the Qubit BR RNA kit, and quality was assessed on the TapeStation 4200 using the RNA ScreenTape assay. The extracted RNA was next utilized for RNA-Seq processing.

### Library preparation and RNA sequencing information

RNA was assessed using the TapeStation (Agilent) and Qubit RNA broad-range assay (Thermofisher). Samples with RINs (RNA Integrity Numbers) of 8 or greater were prepared using the NEBNext Poly(A) mRNA Magnetic Isolation Module (NEB), xGen Broad-range RNA Library Prep (IDT), and xGen Normalase UDI Primer Plate 2 (IDT) where 200ng of total RNA was converted to mRNA using polyA purification. The mRNA is then fragmented and copied into first strand cDNA using reverse transcriptase and random primers. The 3 prime ends of the cDNA are then adenylated and adapters are ligated. The products are purified and enriched by PCR to create the final cDNA library. Final libraries were checked for quality and quantity by Qubit hsDNA (Thermofisher) and LabChip (Perkin Elmer). The samples were sequenced on the Illumina NovaSeq S4 Paired-end 150 bp, according to manufacturer’s recommended protocols.

## Bioinformatics analyses

### Differentially expressed gene analysis

**Fig. 1:**
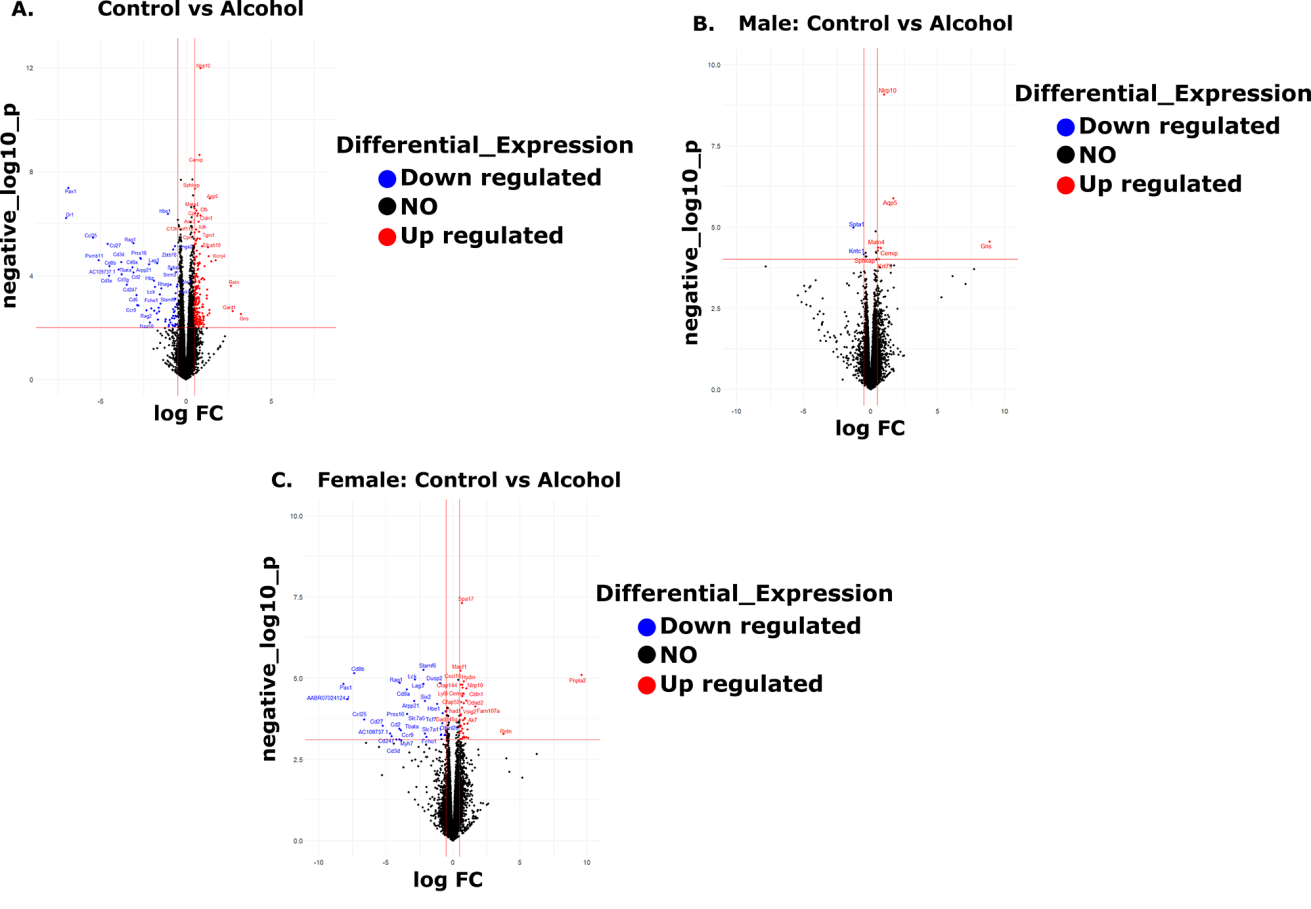
Volcano plots mapping out the distribution of differentially expressed genes (DEGs) in the fetal lungs following alcohol treatment compared with the controls (**a**) independent of sex (Control *n* = 10, Alcohol *n* = 10), (**b**) the male (Control *n* = 5, Alcohol *n* = 5), and (**c**) the female (Control *n* = 5, Alcohol *n* = 5). In all plots (**A-C**), red dots represent genes that are significantly upregulated following alcohol treatment compared to the Controls, blue dots indicate significantly downregulated genes following alcohol treatment compared to the Controls, and black dots represent genes that were not differentially expressed. Significance was established a priori at *P* < 0.05

First, raw bulk RNA-sequenced counts for all samples (*n* = 20) were input to R [29] (version 4.2.2) and transcripts with > 1 count per million across ≥ 3 samples were retained. All differential gene expression analyses comparing samples across treatment and sex were executed using edgeR [30] version 3.40.2’s exact test function. The exact test function returned q-values to control for Type I errors, which are False Discovery Rate-corrected p-values and log 2-fold change (log2FC) values corresponding to the degree of gene expression change between the two groups tested for each pairwise comparison. Positive log2FC values indicate a higher magnitude of transcription for a given gene in the alcohol-treated group (upregulation), while negative log2FC values are indicative of a lower magnitude of gene transcription in the alcohol group (downregulation). Differentially expressed genes (DEGs) are defined as those having a q-value ≤ 0.1 and log2FC ≥|0.5| and are displayed in Fig. 1, which was generated using ggplot2 version 3.4.4 [31]. DEGs were initially annotated with Ensemble IDs and were mapped to rat gene symbols using SYNGO [32], the clusterProfiler R package’s bitr function [33], and the Rat Genome Database [34]. Additionally, all data wrangling in the DEG analysis was executed using dplyr version 1.1.4 [35].

### KEGG pathway ontology analysis

Subsequently, the edgeR DEG output was input to KEGG [36] using the R GAGE package [37] (version 2.48.0) using default parameters. GAGE, using the default two sample t-test returned significantly perturbed KEGG pathways for each pairwise comparison. Significant pathways were also analyzed using ggplot2 version 3.4.4 [31]. The “gene counts” correspond to the number of genes that were tested within the corresponding perturbed KEGG pathway.

### Enrichr-KG network analysis

Post hoc analysis was performed with DEGs that were downregulated in female control versus female alcohol and input to Enrichr-KG were evaluated as described previously [38]. These DEGs were subsequently matched to perturbed terms found in MGI Mammalian Phenotype Level 4 2021 [39], KEGG 2021 Human [36], and GO Biological Process 2021 [40, 41].

### Lung histology

Hematoxylin and eosin (H&E) staining was performed for histopathology assessment of the fetal lungs. Since we did not intend to perform quantitative morphometrics, one male and female pup from a Control and Alcohol dam were used for these assessments. Fetal lungs were dissected, fixed in 10% formalin and embedded in paraffin. Tissue blocks were cut into 5-µm slices using microtome (model RM2235; Leica, Germany). Stained slides were analyzed under a light microscope by a pathologist.

## Results

### Maternal-fetal growth parameters

Dams were sacrificed on GD 20 (Control *n* = 5, Alcohol *n* = 5) and various parameters were measured as shown in Table 1. Maternal weights were not different between the Control and the Alcohol administered dams. Fetal weights of alcohol administered rats (2.09 *±* 0.09) were significantly lower (*P* = 0.0153) compared to the pair-fed control rats (3.05 *±* 0.30) showing a decrease by ~ 37.5%. Crown rump length, was significantly lower with alcohol treatment (*P* = 0.0169) compared to the Controls. Placental efficiency (PE), measured as ratio of fetal weight to placental weight, commonly used as a biomarker of placental function, was significantly different (*P* = 0.0131). There was no difference in litter size between the Control (12.2 *±* 0.80) and Alcohol (12.6 *±* 1.25) dams. The sex ratio, calculated as number of females pups per male was not different between the two groups.

**Table 1:** Maternal and fetal growth parameters and outcomes. Dams were sacrificed on GD 20 (control, *n* = 5; Alcohol, *n* = 5). Values are expressed as Mean *±* SEM. * indicates statistical significance, *P* < 0.05

Measurements	Control	Alcohol	*P*-Value	Significant?
Maternal Weight Start (g)	223.4 *±* 13.66	243.2 *±* 13.39	0.3308	No
Maternal Weight End (g)	324 *±* 8.69	323.1 *±* 18.75	0.9678	No
Maternal Weight Gain (g)	89.11 *±* 17.45	81.28 *±* 6.96	0.6880	No
Litter Size	12.2 *±* 0.80	12.6 *±* 1.25	0.7942	No
Fetal Weight (g)	3.05 *±* 0.30	2.09 *±* 0.09	0.0153*	Yes
Crown-Rump Length (mm)	33.34 *±* 0.66	29.81 *±* 0.97	0.0169*	Yes
Sex-Ratio (M/F)	1.09 *±* 0.20	1.30 *±* 0.29	0.5701	No
Lung Weight/Fetal Weight	0.038 *±* 0.002	0.039 *±* 0.001	0.6960	No
Placental Efficiency	5.00 *±* 0.19	4.18 *±* 0.18	0.0131*	Yes

### Transcriptome analysis

High throughput RNA-sequencing identified a maximum of 14,508 genes in the fetal lungs for comparison. Volcano plots mapping out the distribution of differentially expressed genes (DEGs) in the fetal lungs following alcohol treatment compared with the controls are depicted in Fig. 1. Specifically, the volcano plots depict the log 2-fold change against the log-transformed p-values for the DEGs for each pairwise comparison conducted in edgeR. Log 2-fold change as a function of pairwise comparisons selected based on the treatment and the sex of the rats. Independent of sex, a total of 72 genes were significantly downregulated and 180 genes were upregulated following alcohol exposure in the fetal lungs compared with those in the Controls. These DEGs strongly suggested the existence of a sex-dependent gene regulation dichotomy; in the male lungs only 2 genes were exclusively downregulated and 4 were upregulated compared to the Controls. In contrast, in the female lungs, 34 genes were selectively downregulated and 40 genes were upregulated following in utero alcohol exposure. The difference in number of significantly altered genes when analyzed together versus separated by sex may likely stem from statistical factors including differences in statistical power or pooling effect while doing a combined analysis.

**Fig. 2:**
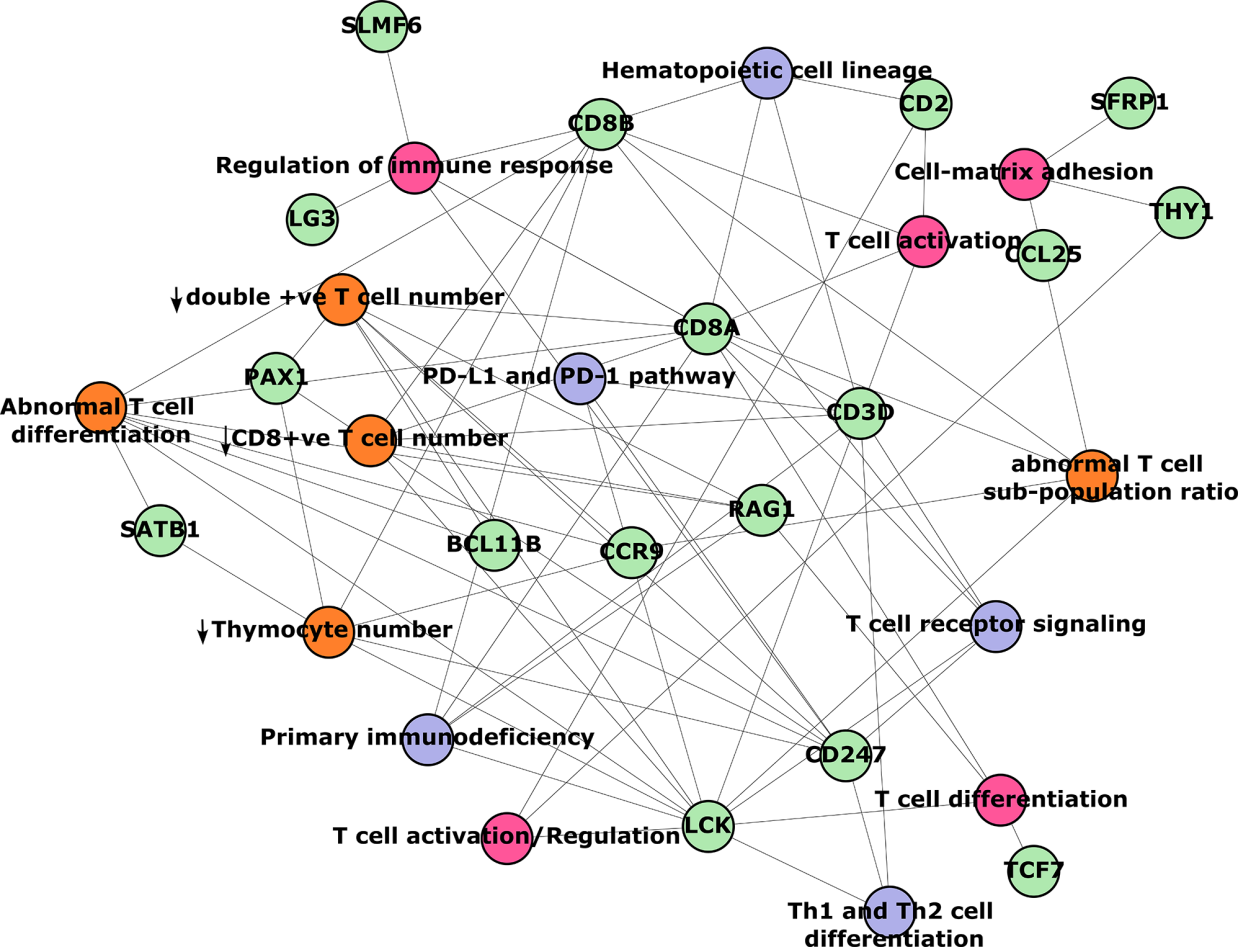
Network analysis plot of downregulated genes in the female fetal lungs following chronic binge alcohol exposure. The nodes are the genes (green circles), while the edges are the significantly perturbed terms found in ontology databases (MGI Mammalian Phenotype Level 4 2021 = orange circles, KEGG 2021 Human = gray circles, and GO Biological Processes 2021 = pink circles). Relationships between nodes and edges are demonstrated by the lines that connect them

A network analysis plot (Fig. 2) of downregulated genes in the female fetal lungs following chronic binge alcohol exposure showed that they were predominantly associated with T cell regulation, notably abnormal T cell regulation, decrease in thymocyte number, a decrease in double positive T cell number, abnormal T cell differentiation, and a decrease in CD8 + T cell number. The nodes depicted in green color are the genes, while the edges are the significantly perturbed terms found in ontology databases, including the MGI Mammalian Phenotype Level 4, KEGG, and GO Biological Processes.

**Fig. 3:**
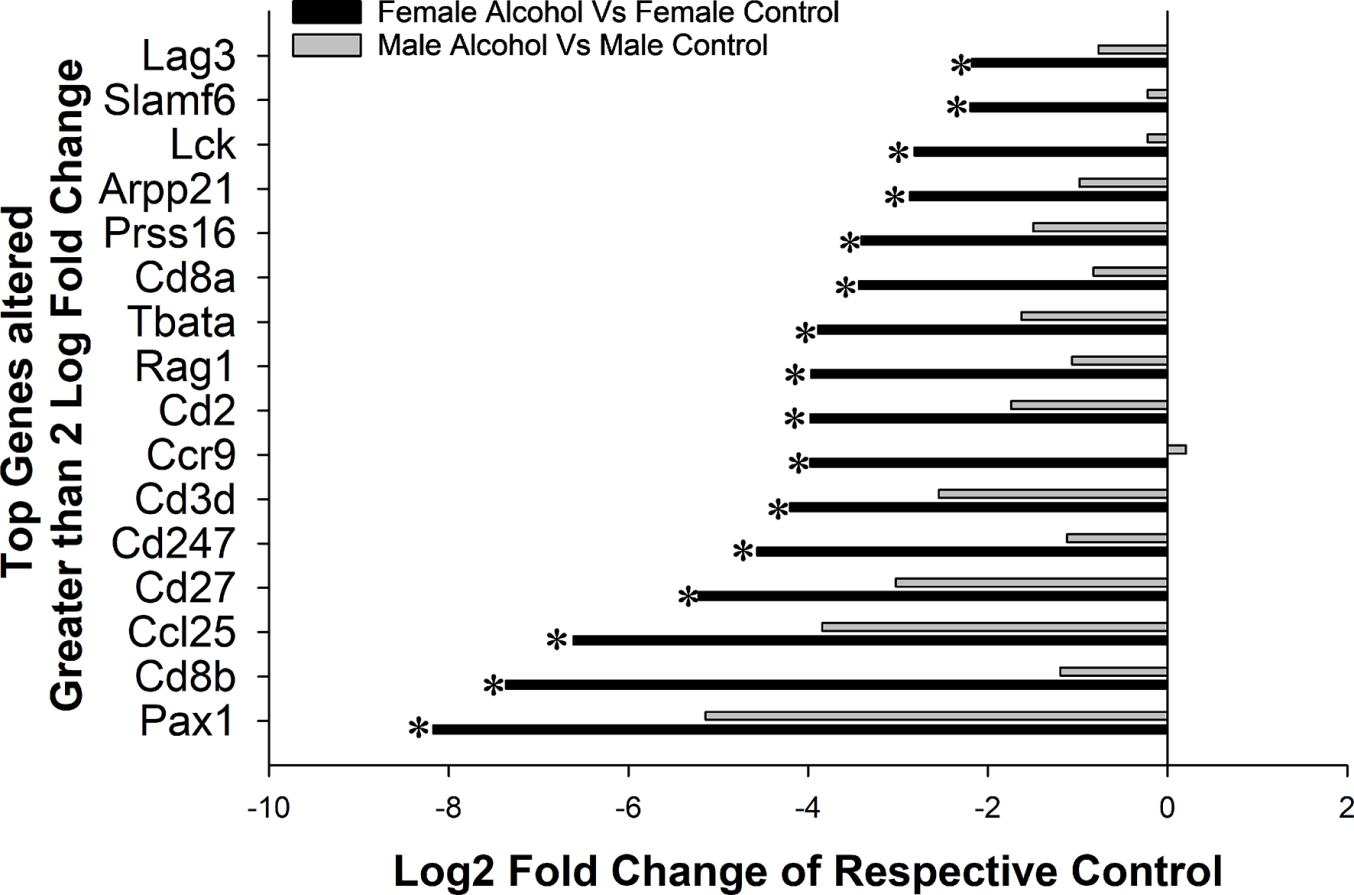
Top genes altered by 2X Log2Fold change in the female and male fetal lungs following chronic binge alcohol exposure compared to their respective Controls. * represents significant change following alcohol treatment compared the respective Controls

The genes that were downregulated in the females were further analyzed; only genes that were altered by greater than a log2 fold change of 2.0 in the females were considered for comparison (Fig. 3). There were a total of 17 genes that were altered by a log2 fold change > 2.15 with a *P* < 0.05 and all of them were downregulated (Fig. 3), except for one gene (RETN) that was upregulated by 3.78 fold following alcohol treatment. The fold change of these 16 genes in the males were also analyzed and notably none of them were significantly downregulated in the male after accounting for false discovery rate. Interestingly, the top genes that were altered were directly related to adaptive immune system associated with the T cell validating the data from the network analysis. The downregulated genes in the female, including Pax1 (↓8.17 fold, *P* = 1.52e-05), Cd8b (↓7.36 fold, *P* = 7.11e-06), Ccl25 (↓6.61 fold, *P* = 0.000188062), Cd27 (↓5.22 fold, *P* = 0.000299549), Cd247 (↓4.56 fold, *P* = 0.000622637), Cd3d (↓4.20 fold, *P* = 0.000778785), Ccr9 (↓3.98 fold, 0.000792526), Cd2 (↓3.98 fold, *P* = 0.000364227), Rag1 (↓3.97 fold, *P* = 1.41e-05), Tbata (↓3.89 fold, *P* = 0.000405168), Cd8a (↓3.43 fold, *P* = 2.25e-05), Prss16 (↓3.41 fold, *P* = 0.000127485), Arpp21 (↓2.87 fold, *P* = 5.09e-05), Lck (↓2.82 fold, *P* = 1.12e-05), Slamf6 (↓2.19 fold, *P* = 5.69e-06), and Lag3 (↓-2.18 fold, *P* = 1.54e-05).

**Fig. 4:**
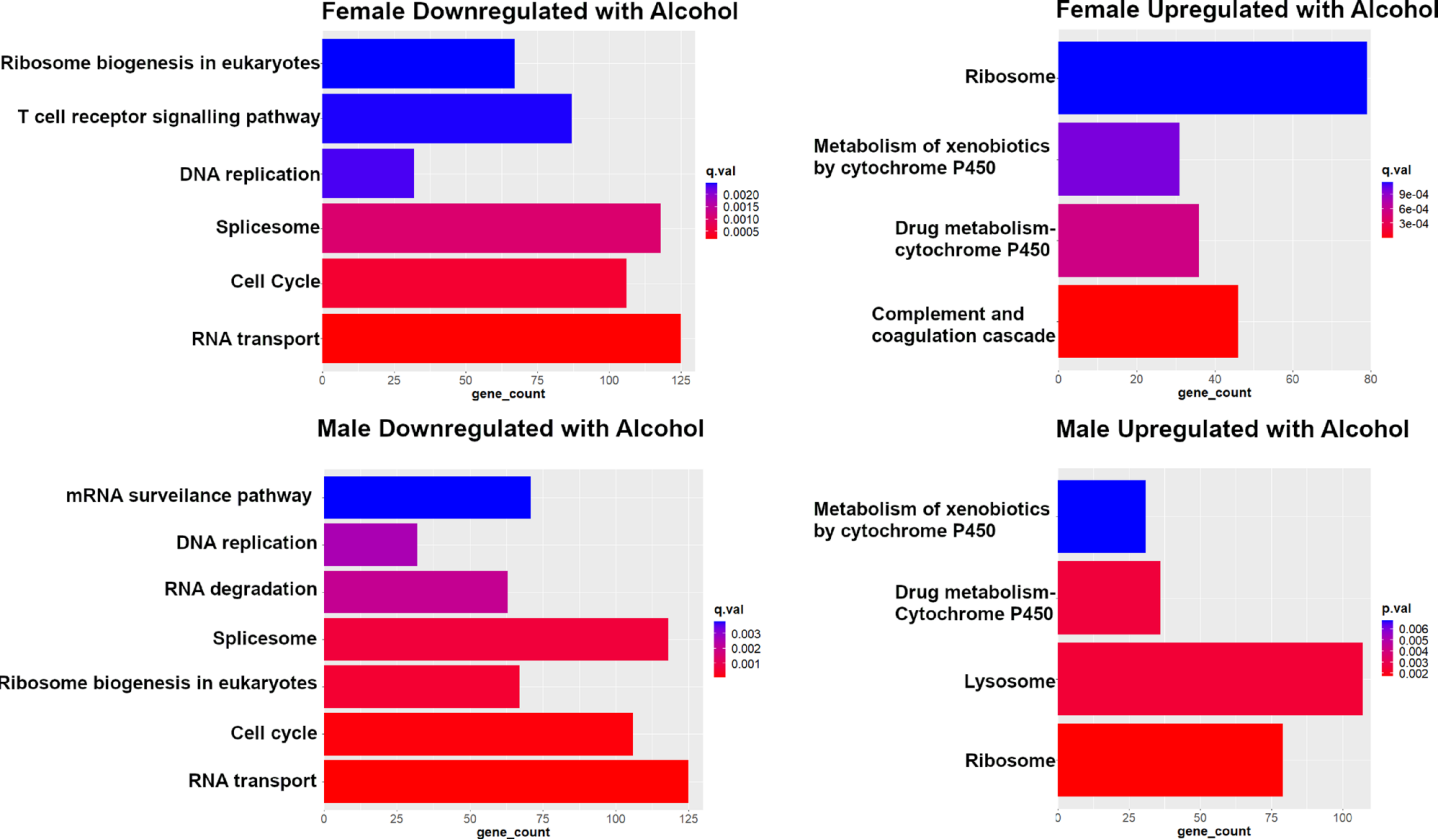
KEGG pathway analyses validate sexual dimorphism associated with alcohol treatment in rats. q-values for all represented pathways are < 0.05

KEGG pathway analyses validated sexual dimorphism associated with alcohol treatment in rats (Fig. 4). The plots demonstrate the number of corresponding genes between KEGG and rat lung RNA sequenced data for the pairwise comparisons of alcohol treated fetal lungs in females compared to female controls and alcohol-treated fetal lungs in males compared to respective male controls. Notably both the males and females exhibited a downregulation in pathways related to cell division and mitosis. Similarly, both the males and females showed an upregulation of pathways related to alcohol metabolism and xenobiotics/metabolism. However, there was a sexual dimorphism in the downregulated genes; the fetal lungs from the females alone exhibited a decrease in T cell signaling and adaptive immune system regulation. A similar downregulation of T cell-related regulation was not found in the males.

**Fig. 5:**
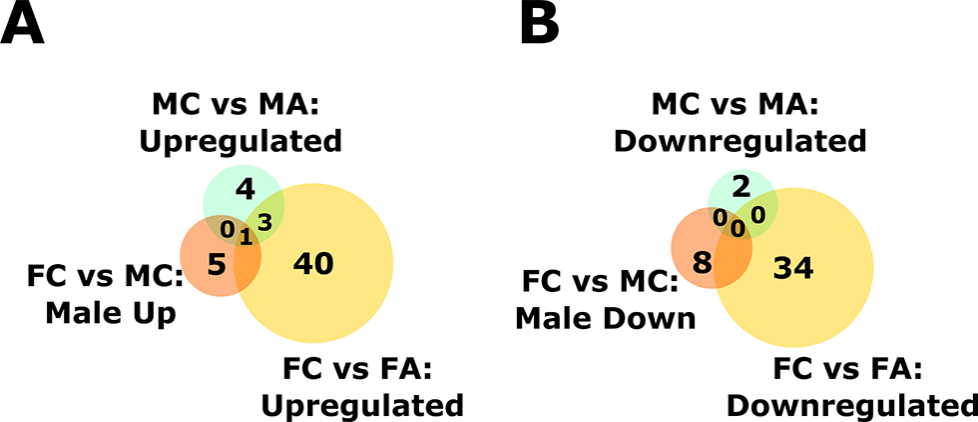
Venn diagrams quantifying the number of common and unique genes that were downregulated and upregulated with alcohol exposure and sex as variables. FC = Female Control, MC = Male Control, FA = Female Alcohol, and MA = Male Alcohol

Venn diagrams were plotted to quantify the common and unique genes that were downregulated and upregulated (Fig. 5). 8 genes were downregulated in the control male lungs compared to the female control lungs. 2 genes were downregulated in the alcohol-exposed fetal male lungs, whereas 34 were downregulated in the females compared to their respective controls. Among the upregulated genes, there were 5 upregulated in the male control lungs compared to the female controls. Only 4 genes were upregulated following alcohol exposure in the males, whereas 40 were upregulated in the females compared to their respective Controls. To validate the RNA-seq data related to a deficit in mitosis and lung development, we performed histopathologic analysis in the fetal lungs from male and female fetuses.

**Fig. 6:**
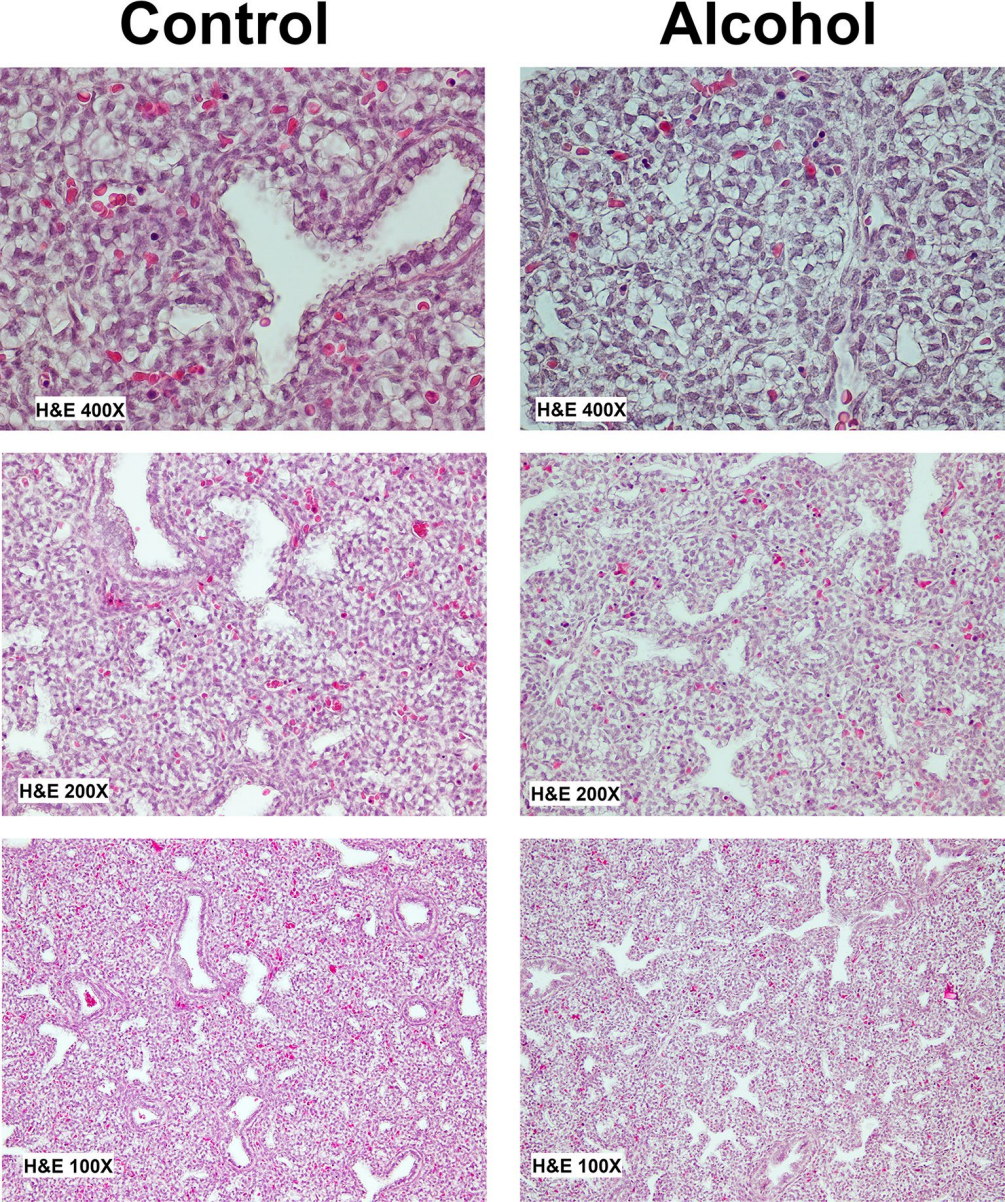
Comparison of gross appearance of fetal lungs following alcohol exposure showed that the developing lungs in both sexes display stunted differentiation following alcohol exposure *in utero.* Representative image is from a male fetal lung

Following alcohol exposure, histopathological analysis was performed on lung tissue. The objective of this analysis was to evaluate and compare the histopathological implications of alcohol exposure on fetal lungs. Comparison of gross appearance and histopathologic morphology showed that both male and female fetal lungs display stunted differentiation (Fig. 6). Further, they were relatively hypoplastic, and displayed a diminution of alveolar size and air spaces. Decreased alveolar capillarization was also evident in the alcohol-exposed fetal lungs in both the sexes.

## Discussion

The novel data from the current study sets the foundation for identification of specific alcohol target pathways in FASD pulmonary phenotype and lung immune etiology. This important endeavor is critical for prevention and development of interventions in children with chronic respiratory conditions or who are susceptible to respiratory infections, particularly if there are aberrations in their pulmonary immune system that may lead to hypersensitive reactions to infections and vaccination. Three principal findings can be gleaned from the current study. First, sexual dimorphism is an important aspect of fetal immune adaptations. Second, sexual dimorphism is an important facet of FASD and FASD immunology. Third, the lungs are an important target in FASD.

### Sexual dimorphism is an important aspect of fetal immune adaptations

The fetal and subsequent neonatal immune system can be shaped by either non-infectious stimuli, such as alcohol consumption, or by infections (viral, bacterial or parasitic) [42]. Inflammation due to viral infections has been associated with increased risk for developmental problems, such as fetal growth restriction [43], autism spectrum disorders (ASD), schizophrenia [44], asthma and allergies [45–50], as well as preterm birth [51]. Previous studies in sheep have also shown a relationship between fetal growth restriction and altered lung development [52, 53]. This study confirms that fetal growth restriction (FGR) is correlated with fetal lung development, but is not able to determine causality. Both male and female subjects exhibited growth restriction and alterations in lung gene expression. However, the female fetuses showed selective decrease in the expression of several genes related to the adaptive immune system, specifically, those related to T cells. The susceptibility of adult females to certain lung diseases and increased severity is well known and develops following adolescence. After childhood, where reproductive hormone expression is considered similar between male and female, it is noted that incidence rates of asthma decrease for males while rates increase for females [54, 55]. Other chronic lung diseases, such a pulmonary arterial hypertension and lymphangioleiomyomatosis predominantly affect females [56], while COPD and bronchiectasis are associated with increased severity in adult females [57, 58]. Autoimmune diseases are also reported to significantly affect females more, including sarcoidosis, systemic sclerosis (SSc), rheumatoid arthritis (RA), Sjogren’s syndrome, and systemic lupus erythematosus, and lung-involvement in these diseases is no different [58]. It has been reported that there is over three fold higher prevalence of lung conditions reported in females compared with males [59]. A greater lifetime exposure to endogenous and exogenous estrogen is associated with more severe asthma [60]. Considering this increased susceptibility of adult female lungs to certain exposures, this study suggests these effects may begin *in utero*.

### Sexual dimorphism is an important facet of FASD and FASD immunology

Unfortunately, there is limited knowledge about alcohol-induced inflammation on the development of both systemic and local (lung mucosal) immune system in the fetus as well as in the neonate. To assess inflammatory/immune adaptations of the fetal lung (mucosal) following chronic binge alcohol consumption, we generated important data using high throughput RNA sequencing. The alcohol exposure period in the current study is during a highly sensitive period, i.e., pregnancy, and both the male and female fetuses are exposed to the same blood alcohol concentration. Another unique feature of this study is that it examines the fetuses prior to delivery rather than examining early childhood. It is documented that female fetuses exhibit a higher regulatory immune response, but male fetuses show a more potent inflammatory response, and this develops into a major dimorphism especially with adaptive T cell response [61]. There is a dearth of information regarding triggers or stressors in pregnancy which causes genetic/environmental/epigenetic changes in the growing fetus, in particular to the establishment of the adult lung immune profile. At least until 2012, Streissguth states that there has been no study that has investigated sex differences in FAS patients, and in FASD work in children do not even specify the number of male vs. female [62]. A recent study in Canada showed a dichotomy in functions affected by alcohol, specifically with neurodevelopment and behavior; females show compromised endocrine regulation, anxiety disorder, and trauma, whereas males show deficits in motor control, ADHD, and problems in school [63]. Similarly, male and female children diagnosed with FASD exhibit altered eye movements with different aspects altered between the sexes compared to their respective Controls [64]. Another recent study with 83 children (ages 6–13) in Canada, showed that females diagnosed with FASD specifically showed the largest perturbations in neural oscillations [65]. One study in a rodent FASD model showed female offspring had lower spleen weight and spleen Tregs, and circulating monocytes were elevated in males [66]. Our data showed prenatal alcohol exposure compromised T Cell related gene expression in females with no significant alteration in males; the downregulated genes in the female, included Pax1, Cd27, Cd247, Cd3d, Ccr9, Cd2, Rag1, Tbata, Cd8a, Prss16, Arpp21, Lck, Slamf6, and Lag3, and all of them were downregulated by > 2 log2fold. In summary, our data shows that there is a clear sexual dimorphism in lung immune system development, and it is important to consider sex as a biological variable while investigating FASD.

### Lung is an important target in FASD

Our data shows that several genes related to lung development were altered following alcohol exposure independent of the sex. Early rodent studies support these findings that second trimester acute alcohol exposure resulted in pulmonary developmental impairments [67]. In sheep, alcohol-exposed fetuses showed significantly decreased surfactant protein (SP)-A mRNA expression and this study suggested increased susceptibility to RSV but did not examine the sexes [68]. Another study in sheep showed prenatal alcohol exposure had significantly reduced fetal lung TNF-α, IL-10, and CCL5 and reduced fetal lung protein level only in preterm lambs [17]. A third study in sheep showed an alcohol-induced decrease in fetal lung IL-1β and IL8 mRNA expression concomitant with an increase in pulmonary collagen 1–α1 and collagen deposition, along with a decrease in SP-A and SP-B mRNA, but again did not examine the sexes [69]. In mice, it was demonstrated that fetal alcohol exposure leads to impaired adaptative immune response with decreased virus-specific lung Cd8 T cells, and a decrease in production of influenza-specific antibodies after an influenza infection [70]. In guinea pigs, prenatal alcohol exposure was demonstrated to impair macrophage function and viability and this was mediated by redox pathways, specifically a decrease in glutathione (GSH) bioavailability [71] that exacerbates the risk for experimentally-induced pneumonia [72, 73]. A review on prenatal alcohol exposure effects on immune-related outcomes including atopic allergies and infection outcomes in the offspring [74], suggests that multiple arms of immune system are affected. Our current study closes a major gap related to the effects of alcohol on the adaptive immune system by classifying sex as an important biological variable.

### Perspectives and significance

The current data forms a strong premise to develop a mechanistic framework for dissecting the contribution of alcohol to lung development, evaluate aberrant postnatal immune response to infections, supporting future studies on inflammatory outcomes in children. The information from the current manuscript will help us understand the immunological mechanisms underlying lung developmental adaptations. This is certain to assist public health policymaking. Toward mechanism, RNA-seq analysis showed a marked and significant response to fetal alcohol exposure at multiple ontological levels consistent with abnormal respiratory development and immune development that was sexually dimorphic. This experiment is the first to provide evidence that gestational binge alcohol consumption can have a direct effect on the transcriptome of the fetal lung in a sexually dimorphic pattern. The current study in conjunction with previous reports of alcohol-induced alterations to the developing lung adaptations by other groups will serve as the base for future investigations and will require further validation by molecular and integrative approaches. The study shows that it is important not to ignore sex as a variable to dissect the subtle changes that arise based on the biological sex.

## Data Availability

No datasets were generated or analysed during the current study.

## References

[1] Wozniak JR, Riley EP, Charness ME. Clinical presentation, diagnosis, and management of fetal alcohol spectrum disorder. Lancet Neurol. 2019;18:760–70. 31160204 10.1016/S1474-4422(19)30150-4 PMC6995665

[2] Caputo C, Wood E, Jabbour L. Impact of fetal alcohol exposure on body systems: a systematic review. Birth Defects Res Part C: Embryo Today: Reviews. 2016;108:174–80. 10.1002/bdrc.21129 27297122

[3] Bertrand JFR, Weber MK, O’Connor M, Riley EP, Johnson KA, Cohen DE. National Task Force on FAS/FAE. Fetal alcohol syndrome: guidelines for referral and diagnosis. Atlanta, GA: Centers for Disease Control and Prevention; 2004.

[4] Popova S, Lange S, Probst C, Gmel G, Rehm J. Estimation of national, regional, and global prevalence of alcohol use during pregnancy and fetal alcohol syndrome: a systematic review and meta-analysis. Lancet Global Health. 2017;5:e290–9. 28089487 10.1016/S2214-109X(17)30021-9

[5] Popova S, Lange S, Probst C, Gmel G, Rehm J. Global prevalence of alcohol use and binge drinking during pregnancy, and fetal alcohol spectrum disorder. Biochem Cell Biol. 2018;96:237–40. 28834683 10.1139/bcb-2017-0077

[6] Popova S, Charness ME, Burd L, Crawford A, Hoyme HE, Mukherjee RA, Riley EP, Elliott EJ. Fetal alcohol spectrum disorders. Nat Reviews Disease Primers. 2023;9:11. 36823161 10.1038/s41572-023-00420-x

[7] D’Alessandro S, Carter L, Webster C. Binge drinking: a review and research agenda. J Consumer Behav. 2023;22:177–98.

[8] Mattson SN, Bernes GA, Doyle LR. Fetal Alcohol Spectrum disorders: a review of the Neurobehavioral deficits Associated with prenatal Alcohol exposure. Alcohol Clin Exp Res. 2019;43:1046–62. 30964197 10.1111/acer.14040 PMC6551289

[9] May PA, Chambers CD, Kalberg WO, Zellner J, Feldman H, Buckley D, Kopald D, Hasken JM, Xu R, Honerkamp-Smith G. Prevalence of fetal alcohol spectrum disorders in 4 US communities. JAMA. 2018;319:474–82. 29411031 10.1001/jama.2017.21896 PMC5839298

[10] Gauthier TW, Drews-Botsch C, Falek A, Coles C, Brown LAS. Maternal alcohol abuse and neonatal infection. Alcoholism: Clin Experimental Res. 2005;29:1035–43. 10.1097/01.alc.0000167956.28160.5e 15976530

[11] Libster R, Ferolla M, Hijano F, Acosta DR, Erviti PL, Polack A, Network FP. Alcohol during pregnancy worsens acute respiratory infections in children. Acta Paediatr. 2015;104:e494–9. 26249835 10.1111/apa.13148

[12] Vilcins D, Blake TL, Sly PD, Saffery R, Ponsonby AL, Burgner D, Tang ML, Reid N, Group BISI. Effects of prenatal alcohol exposure on infant lung function, wheeze, and respiratory infections in Australian children. Alcohol: Clin Experimental Res. 2023;47:2278–87. 10.1111/acer.15205 38151787

[13] Gray D, Willemse L, Visagie A, Czövek D, Nduru P, Vanker A, Stein DJ, Koen N, Sly PD, Hantos Z. Determinants of early-life lung function in African infants. Thorax. 2017;72:445–50. 27856821 10.1136/thoraxjnl-2015-207401 PMC5520243

[14] Gauthier TW. Prenatal Alcohol exposure and the developing Immune System. Alcohol Res. 2015;37:279–85. 26695750 10.35946/arcr.v37.2.11 PMC4590623

[15] Inselman LS, Fisher SE, Spencer H, Atkinson M. Effect of intrauterine ethanol exposure on fetal lung growth. Pediatr Res. 1985;19:12–4. 2578634 10.1203/00006450-198501000-00004

[16] Wang X, Gomutputra P, Wolgemuth DJ, Baxi L. Effects of acute alcohol intoxication in the second trimester of pregnancy on development of the murine fetal lung. Am J Obstet Gynecol. 2007;197:e269261–264. 10.1016/j.ajog.2007.06.031 17826415

[17] Lazic T, Sow FB, Van Geelen A, Meyerholz DK, Gallup JM, Ackermann MR. Exposure to ethanol during the last trimester of pregnancy alters the maturation and immunity of the fetal lung. Alcohol. 2011;45:673–80. 21163613 10.1016/j.alcohol.2010.11.001 PMC3184311

[18] Sozo F, Vela M, Stokes V, Kenna K, Meikle PJ, De Matteo R, Walker D, Brien J, Bocking A, Harding R. Effects of prenatal ethanol exposure on the lungs of postnatal lambs. Am J Physiol Lung Cell Mol Physiol. 2011;300:L139–147. 21036920 10.1152/ajplung.00195.2010 PMC3023283

[19] Gauthier TW, Ping XD, Harris FL, Wong M, Elbahesh H, Brown LA. Fetal alcohol exposure impairs alveolar macrophage function via decreased glutathione availability. Pediatr Res. 2005;57:76–81. 15531743 10.1203/01.PDR.0000149108.44152.D3

[20] Ping XD, Harris FL, Brown LA, Gauthier TW. In vivo dysfunction of the term alveolar macrophage after in utero ethanol exposure. Alcohol Clin Exp Res. 2007;31:308–16. 17250624 10.1111/j.1530-0277.2006.00306.x

[21] Gauthier TW, Ping XD, Gabelaia L, Brown LA. Delayed neonatal lung macrophage differentiation in a mouse model of in utero ethanol exposure. Am J Physiol Lung Cell Mol Physiol. 2010;299:L8–16. 20382747 10.1152/ajplung.90609.2008 PMC2904088

[22] Hause AM, Avadhanula V, Maccato ML, Pinell PM, Bond N, Santarcangelo P, Ferlic-Stark L, Munoz FM, Piedra PA. A cross-sectional surveillance study of the frequency and etiology of acute respiratory illness among pregnant women. J Infect Dis. 2018;218:528–35. 29741642 10.1093/infdis/jiy167 PMC7107407

[23] Klein SL, Hodgson A, Robinson DP. Mechanisms of sex disparities in influenza pathogenesis. J Leukoc Biol. 2012;92:67–73. 22131346 10.1189/jlb.0811427 PMC4046247

[24] Davis-Anderson KL, Wesseling H, Siebert LM, Lunde-Young ER, Naik VD, Steen H, Ramadoss J. Fetal regional brain protein signature in FASD rat model. Reprod Toxicol. 2018;76:84–92. 29408587 10.1016/j.reprotox.2018.01.004 PMC5834402

[25] Subramanian K, Naik VD, Sathishkumar K, Yallampalli C, Saade GR, Hankins GD, Ramadoss J. Chronic binge alcohol exposure during pregnancy impairs rat maternal uterine vascular function. Alcohol Clin Exp Res. 2014;38:1832–8. 24962648 10.1111/acer.12431 PMC4107157

[26] Church MW, Gerkin KP. Hearing disorders in children with fetal alcohol syndrome: findings from case reports. Pediatrics. 1988;82:147–54. 3399287

[27] Cudd TA, Chen W-JA, West JR. Fetal and maternal thyroid hormone responses to ethanol exposure during the third trimester equivalent of Gestation in Sheep. Alcoholism: Clin Experimental Res. 2002;26:53–8. 11821654

[28] Thomas JD, Sather TM, Whinery LA. Voluntary exercise influences behavioral development in rats exposed to alcohol during the neonatal brain growth spurt. Behav Neurosci. 2008;122:1264–73. 19045946 10.1037/a0013271 PMC3164868

[29] Team RC. R: a language and environment for statistical computing. MSOR Connections 2014, 1.

[30] Robinson MD, McCarthy DJ, Smyth GK. edgeR: a Bioconductor package for differential expression analysis of digital gene expression data. Bioinformatics. 2010;26:139–40. 19910308 10.1093/bioinformatics/btp616 PMC2796818

[31] Valero-Mora PM. ggplot2: elegant graphics for data analysis. J Stat Softw Book Reviews. 2010;35:1–3.

[32] Koopmans F, van Nierop P, Andres-Alonso M, Byrnes A, Cijsouw T, Coba MP, Cornelisse LN, Farrell RJ, Goldschmidt HL, Howrigan DP, et al. SynGO: an Evidence-Based, Expert-Curated Knowledge Base for the synapse. Neuron. 2019;103:217–e234214. 31171447 10.1016/j.neuron.2019.05.002 PMC6764089

[33] Yu G, Wang L-G, Han Y, He Q-Y. clusterProfiler: an R Package for comparing Biological themes among Gene clusters. OMICS. 2012;16:284–7. 22455463 10.1089/omi.2011.0118 PMC3339379

[34] Vedi M, Smith JR, Thomas Hayman G, Tutaj M, Brodie KC, De Pons JL, Demos WM, Gibson AC, Kaldunski ML, Lamers L, et al. 2022 updates to the rat genome database: a findable, accessible, interoperable, and Reusable (FAIR) resource. Genetics. 2023;224:iyad042. 36930729 10.1093/genetics/iyad042 PMC10474928

[35] Wickham H, François R. dplyr: A grammar of data manipulation. 2014.

[36] Kanehisa M, Goto S. KEGG: kyoto encyclopedia of genes and genomes. Nucleic Acids Res. 2000;28:27–30. 10592173 10.1093/nar/28.1.27 PMC102409

[37] Luo W, Friedman MS, Shedden K, Hankenson KD, Woolf PJ. GAGE: generally applicable gene set enrichment for pathway analysis. BMC Bioinformatics. 2009;10:161. 19473525 10.1186/1471-2105-10-161 PMC2696452

[38] Chen EY, Tan CM, Kou Y, Duan Q, Wang Z, Meirelles GV, Clark NR. Ma’ayan A: Enrichr: interactive and collaborative HTML5 gene list enrichment analysis tool. BMC Bioinformatics. 2013;14:128. 23586463 10.1186/1471-2105-14-128 PMC3637064

[39] Smith CL, Eppig JT. The mammalian phenotype ontology: enabling robust annotation and comparative analysis. Wiley Interdiscip Rev Syst Biol Med. 2009;1:390–9. 20052305 10.1002/wsbm.44 PMC2801442

[40] Ashburner M, Ball CA, Blake JA, Botstein D, Butler H, Cherry JM, Davis AP, Dolinski K, Dwight SS, Eppig JT, et al. Gene Ontology: tool for the unification of biology. Nat Genet. 2000;25:25–9. 10802651 10.1038/75556 PMC3037419

[41] The Gene Ontology C, Aleksander SA, Balhoff J, Carbon S, Cherry JM, Drabkin HJ, Ebert D, Feuermann M, Gaudet P, Harris NL, et al. The Gene Ontology knowledgebase in 2023. Genetics. 2023;224:iyad031. 36866529 10.1093/genetics/iyad031 PMC10158837

[42] Apostol AC, Jensen KD, Beaudin AE. Training the fetal immune system through maternal inflammation—a layered hygiene hypothesis. Front Immunol. 2020;11:514736. 10.3389/fimmu.2020.00123 PMC7026678 32117273

[43] Shi L, Tu N, Patterson PH. Maternal influenza infection is likely to alter fetal brain development indirectly: the virus is not detected in the fetus. Int J Dev Neurosci. 2005;23:299–305. 15749254 10.1016/j.ijdevneu.2004.05.005

[44] Battle YL, Martin BC, Dorfman JH, Miller LS. Seasonality and infectious disease in schizophrenia: the birth hypothesis revisited. J Psychiatr Res. 1999;33:501–9. 10628526 10.1016/s0022-3956(99)00022-9

[45] Scott NM, Hodyl NA, Osei-Kumah A, Stark MJ, Smith R, Clifton VL. The presence of maternal asthma during pregnancy suppresses the placental pro-inflammatory response to an immune challenge in vitro. Placenta. 2011;32:454–61. 21453968 10.1016/j.placenta.2011.03.004

[46] Lotvall J, Ekerljung L, Lundback B. Multi-symptom asthma is closely related to nasal blockage, rhinorrhea and symptoms of chronic rhinosinusitis-evidence from the West Sweden Asthma Study. Respir Res. 2010;11:163. 21110834 10.1186/1465-9921-11-163 PMC3004848

[47] Akbari O, Stock P, Singh AK, Lombardi V, Lee WL, Freeman GJ, Sharpe AH, Umetsu DT, Dekruyff RH. PD-L1 and PD-L2 modulate airway inflammation and iNKT-cell-dependent airway hyperreactivity in opposing directions. Mucosal Immunol. 2010;3:81–91. 19741598 10.1038/mi.2009.112 PMC2845714

[48] Subbarao P, Mandhane PJ, Sears MR. Asthma: epidemiology, etiology and risk factors. CMAJ: Can Med Association J = J de l’Association medicale canadienne. 2009;181:E181–190. 10.1503/cmaj.080612 PMC2764772 19752106

[49] Hammad H, Chieppa M, Perros F, Willart MA, Germain RN, Lambrecht BN. House dust mite allergen induces asthma via toll-like receptor 4 triggering of airway structural cells. Nat Med. 2009;15:410–6. 19330007 10.1038/nm.1946 PMC2789255

[50] Borrego LM, Arroz MJ, Videira P, Martins C, Guimaraes H, Nunes G, Papoila AL, Trindade H. Regulatory cells, cytokine pattern and clinical risk factors for asthma in infants and young children with recurrent wheeze. Clin Exp Allergy. 2009;39:1160–9. 19438590 10.1111/j.1365-2222.2009.03253.x

[51] Cappelletti M, Della Bella S, Ferrazzi E, Mavilio D, Divanovic S. Inflammation and preterm birth. J Leukoc Biol. 2016;99:67–78. 26538528 10.1189/jlb.3MR0615-272RR

[52] Maritz GS, Cock CA, Louey S, Joyce BJ, Albuquerque CA, Harding R. Effects of fetal growth restriction on lung development before and after birth: a morphometric analysis. Pediatr Pulmonol. 2001;32:201–10. 11536449 10.1002/ppul.1109

[53] Maritz GS, Cock ML, Louey S, Suzuki K, Harding R. Fetal growth restriction has long-term effects on postnatal lung structure in sheep. Pediatr Res. 2004;55:287–95. 14630984 10.1203/01.PDR.0000106314.99930.65

[54] Johnson C, Havstad S, Ownby D. Children’s respiratory and Environmental Workgroup in the ECHO Consortium. Pediatric Asthma incidence rates in the United States from 1980 to 2017. J Allergy Clin Immunol. 2021;148:1270–80. 33964299 10.1016/j.jaci.2021.04.027 PMC8631308

[55] Pignataro F, Bonini M, Forgione A, Melandri S, Usmani O. Asthma and gender: the female lung. Pharmacol Res. 2017;119:384–90. 28238829 10.1016/j.phrs.2017.02.017

[56] Taveira-DaSilva AM, Moss J. Optimizing treatments for lymphangioleiomyomatosis. Expert Rev Respir Med. 2012;6:267–76. 22788941 10.1586/ers.12.26 PMC3429940

[57] Townsend EA, Miller VM, Prakash Y. Sex differences and sex steroids in lung health and disease. Endocr Rev. 2012;33:1–47. 22240244 10.1210/er.2010-0031 PMC3365843

[58] Vink NM, Postma DS, Schouten JP, Rosmalen JG, Boezen HM. Gender differences in asthma development and remission during transition through puberty: the TRacking adolescents’ individual lives Survey (TRAILS) study. J Allergy Clin Immunol. 2010;126:498–504. e496. 20816186 10.1016/j.jaci.2010.06.018

[59] Lachowicz-Scroggins ME, Vuga LJ, Laposky AD, Brown M, Banerjee K, Croxton TL, Kiley JP. The intersection of women’s health, lung health, and disease. Volume 321. American Physiological Society Rockville, MD. 2021. p. L624. 10.1152/ajplung.00333.2021 PMC8461798 34431414

[60] Zein JG, Erzurum SC. Asthma is different in women. Curr Allergy Asthma Rep. 2015;15:1–10. 10.1007/s11882-015-0528-y PMC4572514 26141573

[61] Baines KJ, West RC. Sex differences in innate and adaptive immunity impact fetal, placental, and maternal health. Biol Reprod. 2023;109:256–70. 37418168 10.1093/biolre/ioad072

[62] Streissguth AP. Sex differences in prenatal alcohol abuse in humans. 2012.

[63] Flannigan K, Poole N, Cook J, Unsworth K. Sex-related differences among individuals assessed for fetal alcohol spectrum disorder in Canada. Alcohol: Clin Experimental Res. 2023;47:613–23. 10.1111/acer.15017 36932990

[64] Paolozza A, Munn R, Munoz DP, Reynolds JN. Eye movements reveal sexually dimorphic deficits in children with fetal alcohol spectrum disorder. Front NeuroSci. 2015;9:76. 25814922 10.3389/fnins.2015.00076 PMC4356081

[65] Candelaria-Cook FT, Schendel ME, Romero LL, Cerros C, Hill DE, Stephen JM. Sex-specific differences in Resting Oscillatory Dynamics in children with prenatal Alcohol exposure. Neuroscience. 2024;543:121–36. 38387734 10.1016/j.neuroscience.2024.02.016 PMC10954390

[66] Bake S, Pinson MR, Pandey S, Chambers JP, Mota R, Fairchild AE, Miranda RC, Sohrabji F. Prenatal alcohol-induced sex differences in immune, metabolic and neurobehavioral outcomes in adult rats. Brain Behav Immun. 2021;98:86–100. 34390803 10.1016/j.bbi.2021.08.207 PMC8591773

[67] Wang X, Gomutputra P, Wolgemuth DJ, Baxi L. Effects of acute alcohol intoxication in the second trimester of pregnancy on development of the murine fetal lung. Am J Obstet Gynecol. 2007;197:269. e261-269. e264. 10.1016/j.ajog.2007.06.031 17826415

[68] Lazic T, Wyatt TA, Matic M, Meyerholz DK, Grubor B, Gallup JM, Kersting KW, Imerman PM, Almeida-De-Macedo M, Ackermann MR. Maternal alcohol ingestion reduces surfactant protein A expression by preterm fetal lung epithelia. Alcohol. 2007;41:347–55. 17889311 10.1016/j.alcohol.2007.07.006 PMC2083706

[69] Sozo F, O’Day L, Maritz G, Kenna K, Stacy V, Brew N, Walker D, Bocking A, Brien J, Harding R. Repeated ethanol exposure during late gestation alters the maturation and innate immune status of the ovine fetal lung. Am J Physiology-Lung Cell Mol Physiol. 2009;296:L510–8. 10.1152/ajplung.90532.2008 19112099

[70] McGill J, Meyerholz DK, Edsen-Moore M, Young B, Coleman RA, Schlueter AJ, Waldschmidt TJ, Cook RT, Legge KL. Fetal exposure to ethanol has long-term effects on the severity of influenza virus infections. J Immunol. 2009;182:7803–8. 19494304 10.4049/jimmunol.0803881 PMC2692078

[71] Gauthier TW, Ping X-D, Harris FL, Wong M, Elbahesh H, Brown LAS. Fetal alcohol exposure impairs alveolar macrophage function via decreased glutathione availability. Pediatr Res. 2005;57:76–81. 15531743 10.1203/01.PDR.0000149108.44152.D3

[72] Gauthier TW, Young PA, Gabelaia L, Tang SM, Ping XD, Harris FL, Brown LAS. In utero ethanol exposure impairs defenses against experimental group B streptococcus in the term Guinea pig lung. Alcoholism: Clin Experimental Res. 2009;33:300–6. 10.1111/j.1530-0277.2008.00833.x PMC2662374 19032578

[73] Ping XD, Harris FL, Brown LAS, Gauthier TW. In vivo dysfunction of the term alveolar macrophage after in utero ethanol exposure. Alcoholism: Clin Experimental Res. 2007;31:308–16. 10.1111/j.1530-0277.2006.00306.x 17250624

[74] Reid N, Moritz KM, Akison LK. Adverse health outcomes associated with fetal alcohol exposure: a systematic review focused on immune-related outcomes. Pediatr Allergy Immunol. 2019;30:698–707. 31215695 10.1111/pai.13099

